# Evaluation of the effectiveness of local anesthesia approaches for symptomatic irreversible pulpitis: a systematic review and meta-analysis

**DOI:** 10.3389/fdmed.2025.1679706

**Published:** 2026-01-14

**Authors:** Xu Li, Xin Chen, Qian Wang, Yuqing Gui, Fengqing Huang, Dinghao Zhong, Lijun Xiong, Mengyan Xiao, Zining Luo, Junxian Gu, Xinyu Xu, Jiebin Xie

**Affiliations:** 1 Department of Gastrointestinal Surgery, Affiliated Hospital of North Sichuan Medical College, Sichuan, Nanchong, China; 2 School of Clinical Medicine, North Sichuan Medical College, Sichuan, Nanchong, China; 3 School of Imaging, North Sichuan Medical College, Sichuan, Nanchong, China; 4 School of Stomatology, North Sichuan Medical College, Sichuan, Nanchong, China

**Keywords:** anesthesia, articaine, inferior alveolar nerve block (IANB), meta-analysis, symptomatic irreversible pulpitis (SIP)

## Abstract

**Objective:**

This meta-analysis assessed the efficacy of various anesthetic protocols for symptomatic irreversible pulpitis, comparing techniques and agents to identify the optimal anesthesia approach.

**Methods:**

We conducted a comprehensive search of the Cochrane Library, PubMed, Web of Science, Scopus, and Embase databases up to July 10, 2025, identifying relevant studies based on predefined inclusion and exclusion criteria. The primary outcome was the success rate of anesthesia. Data extraction and quality assessment were performed using a pre-designed form and the revised Cochrane Risk of Bias Tool. A fixed-effect model was used for meta-analysis when heterogeneity was low (*I*^2^ ≤ 50%, *p* ≥ 0.1); otherwise, a random-effects model was adopted. Additionally, another model was employed for validation, and the results from both models were compared to derive more reasonable conclusions. Publication bias was assessed using funnel plots and the Egger test.

**Results:**

Fourteen RCTs were included in the meta-analysis. Pooled analysis showed that modified anesthetic protocols for SIP were 3.62 times more successful than conventional inferior alveolar nerve block (IANB) using standard 2% lidocaine with epinephrine (OR = 3.34; 95% CI: 2.49–4.48). Studies conducted in Iran had the highest success rate (OR = 4.31; 95% CI: 3.59–5.17, *p* < 0.001). Inferior alveolar nerve block (IANB) was more effective than buccal infiltration (OR = 4.03; 95% CI: 3.38–4.81, *p* < 0.001), and 4% articaine demonstrated the highest efficacy (OR = 4.18; 95% CI: 2.85–6.16, *p* < 0.001).

**Conclusion:**

This meta-analysis assessed the efficacy of various anesthetic protocols for SIP, comparing techniques and agents to identify the optimal anesthesia approach.

**Systematic Review Registration:**

https://www.crd.york.ac.uk/PROSPERO/recorddashboard, PROSPERO database CRD42025638427.

## Introduction

Symptomatic irreversible pulpitis (SIP) is an acute, intensely painful inflammatory condition of the dental pulp and constitutes one of the principal emergencies encountered in daily dental practice (1, 2). The predominant aetiologies—deep carious lesions or traumatic exposure—facilitate bacterial invasion, triggering an irreversible cycle of pulpal ischaemia, necrosis, and subsequent periapical pathosis (3, 4). If left untreated, it can lead to pulp necrosis and periapical disease, tooth mobility, and even tooth loss (5–7). The global incidence of pulpitis is about 30%, which imposes a substantial economic burden on individuals, medical institutions, and the government (8). Achieving effective local anesthesia is a key step in the treatment of SIP and a necessary condition for reducing patient discomfort (9). Nevertheless, achieving consistently effective pulpal anesthesia in the presence of active inflammation remains a formidable clinical challenge.

Two commonly used mandibular anesthesia techniques for the treatment of SIP are IANB and BI (10). IANB has been the preferred technique for anesthetizing SIP (11). Studies indicate that the failure rate of IANB is approximately 12%–65% (12–15). Major influencing factors include decreased local pH, activation, and sensitization of nociceptive neurons, the action of inflammatory mediators, collateral innervation, and rapid drug tolerance (16–18). Moreover, IANB may be accompanied by varying degrees of complications, including pain, trismus, hematoma, ptosis, and facial nerve palsy (19–22). The success of buccal infiltration is influenced by factors such as tooth location, root length, severity of inflammation, and the anesthetic used. Studies by Askari et al. (23) showed that longer root lengths are associated with a higher failure rate of BI. Therefore, selecting an appropriate anesthetic protocol is crucial for ensuring the success of dental procedures.

Recent investigations have pursued multimodal strategies to overcome anaesthetic failure in inflamed pulps. Adjunctive techniques—including lingual infiltration (LI), intraligamentary (ILI), and intraosseous (IOI) injection—have been evaluated, together with pre-operative pharmacological modulation using non-steroidal anti-inflammatory drugs (NSAIDs) or systemic corticosteroids (23). For example, a randomized clinical trial showed that preoperative oral administration of nonsteroidal anti-inflammatory drugs or corticosteroids significantly improved the success rate of IANB in patients with SIP. Among the commonly used anesthetics, 2% lidocaine and 4% articaine are widely used (24). Moreover, the choice of an anesthetic also plays a crucial role. The thiophene ring in articaine has higher lipophilicity than the benzene ring in lidocaine, allowing it to penetrate tissue more efficiently than 2% lidocaine (25, 26). This gives the articaine an advantage in the treatment of severe pulpitis that requires a stronger anesthetic. Selecting the appropriate anesthetic dose is also considered a way to improve the success rate of anesthesia (27). In addition, additives in anesthetics also affect the success rate of anesthesia. Adrenaline is often added to anesthetics to prolong their action by constricting blood vessels, reducing systemic absorption, and reducing the risk of toxicity (28). To add to this, other additives, such as mannitol and sodium bicarbonate, can enhance the efficacy of IANB (29, 30).

In this meta-analysis, “conventional treatment” refers to standard IANB using 2% lidocaine with epinephrine (1:80,000–1:200,000), administered as a single-technique approach without supplementary injections or pharmacological modifications. Previous studies found that 4% articaine had a higher success rate than 2% lidocaine (31–34); however, Kung et al. (35) and Brandt et al. (33) found no difference in IANB anesthesia success rates between lidocaine and articaine. Mepivacaine and lidocaine have similar anesthetic effects on IANB in patients with symptomatic irreversible pulpitis (36). Using a larger volume of local anesthetic can improve the success rate of IANB (27). Conversely, a randomized clinical trial (37) reported no significant difference in the success rate of IANB when the volume of local anesthetic solution was increased. In summary, the current literature and existing evidence are contradictory. Moreover, although some systematic reviews have considered the addition of additives (sodium bicarbonate) to lidocaine solutions for anesthesia in endodontics (38, 39), no comprehensive study has considered all additives. Due to the diverse components and outcomes of additives, current systematic studies have not fully considered the impact of all possible anesthetic protocols on the success rate of mandibular anesthesia.

Once anesthesia fails, supplementary techniques are necessary. However, these additional procedures subject patients to further stress, exacerbating the pain associated with dental treatment and the anesthesia itself (40). Therefore, it is more reasonable to establish a painless anesthesia protocol from the outset (41). This systematic review aims to synthesize the latest clinical evidence, explore the effectiveness of current anesthesia protocols, and evaluate the best anesthesia techniques and drugs for dental surgery to provide clinicians with clear guidance to improve patient outcomes and increase the success rate of local anesthesia in root canal clinics.

## Methods

### Protocol and registration

The present meta-analysis was performed according to the Preferred Reporting Items for Systematic Reviews and Meta-Analyses Guidelines for systematic reviews and meta-analyses (42). The study was prospectively designed and registered in advance with the International Prospective Register of Systematic Reviews (PROSPERO) under the registration number CRD42025638427.

### Inclusion criteria and exclusion criteria

The inclusion criteria were created based on the PICOS strategy (PRISMA) (42). The eligibility criteria were as follows:

1. Population: Adults (≥18 years) with SIP confirmed by cold test (Endo-Ice or CO_2_ snow) and electric pulp testing (EPT).
2. Interventions: The main methods of anesthesia include buccal infiltration, tongue infiltration, instrumental injection, interseptal injection, or a combination of these techniques (anesthesia techniques must be clearly defined and reproducible), including: Buccal infiltration (BI): 1.8 mL of 4% articaine with 1:100,000 epinephrine via 30-gauge needle at the mucobuccal fold adjacent to the target tooth. Lingual infiltration (LI): 0.9 mL of the same anesthetic at the lingual gingiva of the target tooth. Intraligamentary injection (PDL): 0.2 mL per root via 27-gauge needle using computer-controlled local anesthetic delivery (CCLAD). Intraseptal injection (IS): 0.3 mL at the center of the interdental papilla with a 25-gauge needle. Combination techniques must be explicitly described (e.g., BI + LI, BI + PDL).
3. Control: IANB (local anesthetic drugs were injected into the mandibular foramen on the medial side of the ascending branch of the mandible to block the conduction of the inferior alveolar nerve before entering the mandibular foramen, thus achieving the anesthetic effect).
4. Outcomes: the success rate of anesthesia, defined as the proportion of patients achieving adequate pain control and procedural comfort during dental interventions.
5. Study design: Only parallel-group, triple-blind (patient, operator, evaluator) randomized controlled trials (RCTs).

The exclusion criteria were: 1) Letters, observational study, report cases, and case series. 2) Studies that reported including patients on the use of any medications that may influence pain assessment, such as analgesics, anti-inflammatories, sedatives, antidepressants, or anxiolytics. 3) Articles with insufficient or unavailable data. 4) Studies that did not report results in English. 5) Duplications of publications.

### Search approach

We developed a robust search strategy based on established methods from previous meta-analyses to ensure a comprehensive and systematic approach. We conducted a thorough literature search in the Cochrane Library, PubMed, Scopus, Web of Science, and Embase databases up to July 10, 2025. Our search terms included medical subject headings (MeSH) and relevant keywords such as “Pulpitis,” “Anesthetics,” “Inflammation,” “Endodontic,” “Anesthetic,” “Anesthetic Drugs,” and “Drugs, Anesthetic.” We utilized Note Express (v4.1.0.10030) software for citation management to streamline the retrieval process and eliminate duplicate records. When multiple publications reported the same study, we prioritized the most complete and up-to-date version for inclusion. The full search strategy for each database can be found in Supplementary Tables 1–3.

The study selection process was conducted with meticulous attention to detail by a panel of three reviewers (A, B, and C), following predefined inclusion and exclusion criteria. Each reviewer independently performed an initial screening of the retrieved literature, eliminating duplicates and articles deemed irrelevant based on their titles and abstracts. Subsequently, the full texts of articles that appeared potentially eligible were obtained and subjected to a thorough review to ensure the comprehensive inclusion of all relevant research. Two examiners (A and B) independently conducted the data extraction phase. Upon completion, a cross-validation process was performed, with discrepancies resolved through consultation with the third examiner D. The findings were then discussed among the three reviewers to reach a consensus, thereby ensuring the robustness and reliability of the study selection and data extraction processes.

### Data extraction

By referring to the data extracted from previously published meta-analyses (43–46) and combining it with the characteristics of this study, we designed a data extraction table to carefully extract the following comprehensive data: study type (specifically randomized controlled trials, RCTs), the first author's name, the year of publication, the geographical regions of the patients involved, detailed characteristics of the participants, the sample size of the intervention group, the sample size of the control group, the specific type of anesthesia administered, the precise definition of pulpitis utilized in the study, the classification of the intervention, the definition of the primary outcome measure, the age of participants at the baseline assessment, and the calculated value of the outcome variable, expressed as an odds ratio.

### Quality assessment

Two reviewers (A and B) independently assessed the risk of bias in the included studies using the revised Cochrane Risk of Bias Tool for Randomized Trials. This tool is structured into five domains, each addressing a specific source of potential bias: (1) bias arising from the randomization process; (2) bias due to deviations from intended interventions; (3) bias due to missing outcome data; (4) bias in the measurement of the outcome; and (5) bias in the selection of the reported result. Each domain was evaluated using signaling questions, with the risk of bias categorized as low (green), some concerns (yellow), or high (red) based on the responses.

Discrepancies in the risk of bias assessments were resolved through consultation with a third reviewer, C.

Finally, the risk of bias graph was generated using Review Manager 5.4 by reviewer A to visually summarize the bias assessments across the included studies.

### Outcome indicators

The primary outcome indicator in this study was the success rate of anesthesia, defined as the proportion of patients achieving adequate pain control and procedural comfort during dental interventions. Successful anesthesia is defined as the patient's ability to obtain adequate pain control during treatment, and the patient's vital signs, such as blood pressure and heart rate, remain stable during anesthesia without a significant stress response.

### Statistical analysis

In this meta-analysis, data were meticulously extracted from each study. Given that the primary outcome was the success rate of anesthesia (defined as whether the patient achieved adequate pain control during the dental procedure), which is a binary variable, effect sizes describing continuous variables such as SMD were not selected, considering that relative risk (RR) is generally only applicable to prospective studies and is mathematically asymmetrical and less stable. Therefore, choosing the odds ratio (OR) as the measure of effect size is appropriate. Heterogeneity among the included studies was assessed using the Cochrane *Q*-*p* value and the *I*^2^ statistical heterogeneity test. A fixed-effect model was employed when heterogeneity was not significant (*I*^2^ ≤ 50%, *p* ≥ 0.1). In cases where heterogeneity was present, sensitivity analysis was conducted to identify and eliminate potential sources of heterogeneity (refer to the explanation of *I*^2^ provided in the Cochrane Handbook 11.3.2). Specifically, studies were sequentially excluded to determine the source of heterogeneity, and the heterogeneity was further analyzed. If heterogeneity remained substantial after sensitivity analysis, a random-effects model was adopted. Additionally, the other model was employed for validation, and the results from both models were compared to derive more reasonable conclusions.

To assess potential publication bias in our meta-analysis, we first employed the Egger test with a significance level set at *p* < 0.05, indicating potential publication bias. If such a bias was suggested, we planned to conduct a more detailed investigation. To address potential publication bias, we utilized the trim-and-fill method as a supplementary approach following the initial evaluation with funnel plots and the Egger test. This method was selected for its capability to estimate and adjust for missing studies, particularly in meta-analyses with a limited number of included studies.

The trim-and-fill method comprises three key steps:

Trimming: Studies contributing to funnel plot asymmetry were identified and temporarily omitted to generate a symmetric funnel plot. This step helps to isolate studies that may be indicative of publication bias.

Filling: Hypothetical missing studies were imputed on the opposite side of the funnel plot to restore symmetry. The number and location of these imputed studies were determined based on the assumption that the funnel plot should be symmetric without bias.

Re-estimation: The pooled effect size was recalculated using the adjusted dataset, which incorporated both the original studies and the imputed missing studies. This adjusted effect size provides an estimate of the overall effect after accounting for potential publication bias.

This rigorous approach ensures the robustness and reliability of the meta-analysis results, even when potential publication bias is present. For the comprehensive data analysis, the advanced statistical software Stata, version 17.0, and Review Manager 5.4 were harnessed to ensure robust and reliable results. Other software is also involved, including Note Express (v4.1.0.10030).

## Results

### Description of the included studies

**Figure 1:**
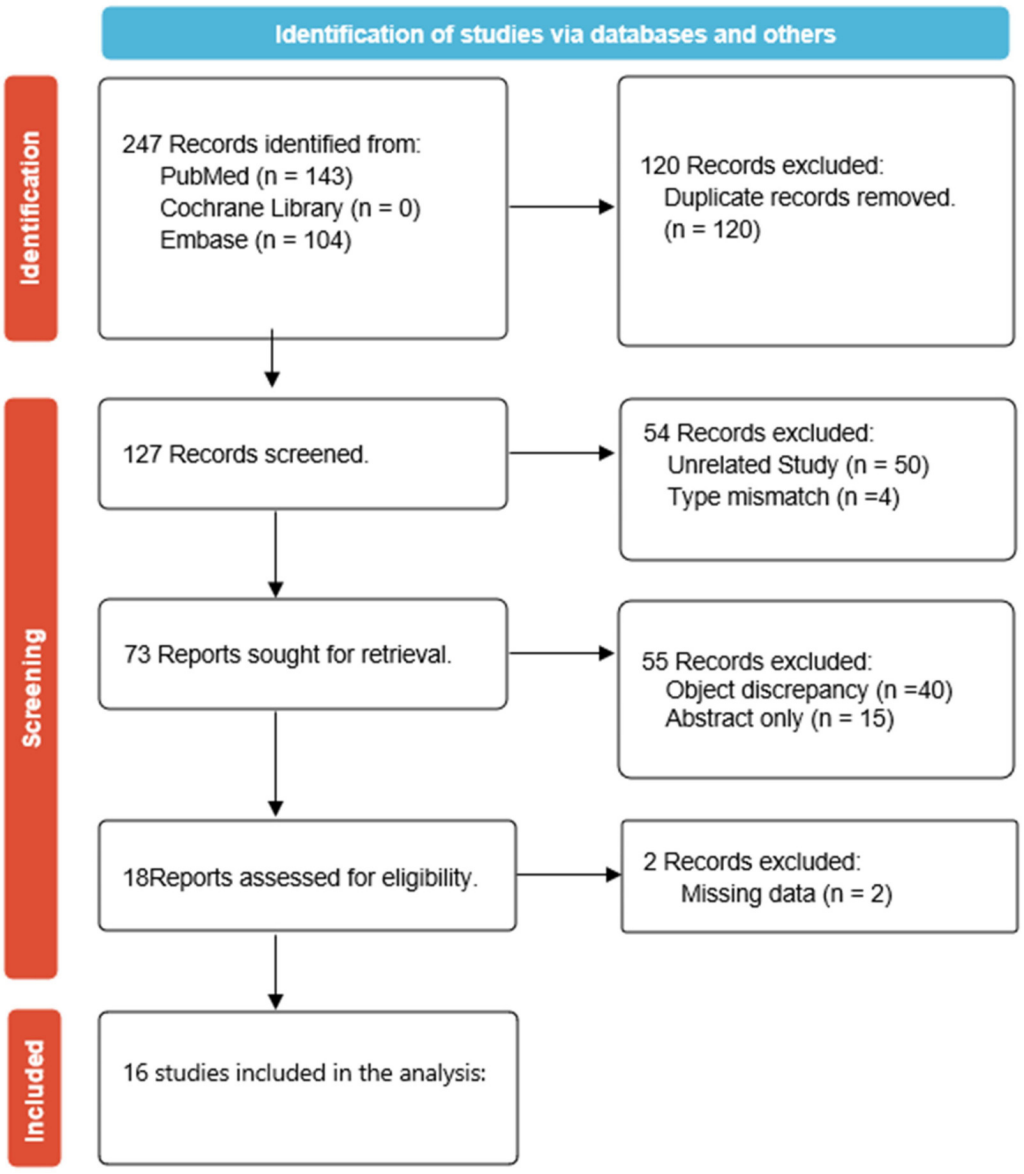
Preferred reporting items for systematic reviews and meta-analysis (PRISMA) flow diagram summarizing the search strategy and study selection for the meta-analysis.

A total of 247 articles were identified during our search. Of these, 146 were from PubMed, 0 from Cochrane Library, 1167 from Scopus, 63 from Web of Science, and 104 from Embase. After the first round of screening, a total of 120 papers with duplicates and topics not related to pulpitis were excluded. And then a second round of screening. 54 unrelated articles were deleted. The process is shown in Figure 1. Finally, a total of 16 studies (9, 27, 47–60), including 16 randomized controlled trials, were included after consensus. The characteristics of the 16 studies are listed in Table 1

**Table 1:** Summary of main characteristics of the 16 included studies.

NO	Type	Author	Year	Areas	Characteristics of participants	Number of the Intervention group	Number of the control group	Anesthesia (definition)	Intervene classification	Outcome	Age at baseline	Key conclusions
1	RCT	Michael R. Shapiro	2018	US	Meet the accepted American Association of Endodontics diagnostic criteria for a mandibular first or second molar with symptomatic IP	36	35	Articaine, Lidocaine	First molars BI receive 4% articaine First molars BI receive 2% lidocaine Second molars BI receive 4% articaine Second molars BI receive 2% lidocaine	Anaesthesia success	24–54	Intranasal ketorolac offers no significant advantage over N_2_O/O_2_ alone; supplemental anesthesia remains necessary
2	RCT	Shrimanikandan	2021	India	Vital mandibular molar tooth with moderate to severe active pain and a prolonged response to cold testing with an ice stick, and an electric pulp tester (Parkell, D624, Farmingdale, NY 11735, USA®) with vital coronal pulp tissue on access opening	30	30	Articaine, Lidocaine	Group I: Thirty patients received IANB of 2% lidocaine without buccal infiltration. Group II: Thirty patients received IANB of 2% lidocaine followed by buccal infiltration with 2% lidocaine. Group III: Thirty patients received IANB with 4% articaine followed by buccal infiltration with 4% articaine.	Anaesthesia success	21–38	Articaine dual-injection protocol significantly outperforms lidocaine but still falls short of universal efficacy
3	RCT	Omid Dianat	2019	Iran	All teeth had fully formed roots (based on periapical radiographs). Clinical diagnosis of symptomatic irreversible pulpitis was confirmed by a report of recent spontaneous pain of the involved molar, positive response to electric pulp test (Sybron Endo, Kerr, Italy), and a moderate to severe lingering pain to cold test using Roeko Endo-Frost	30	30	supplemental intraseptal and buccal infiltration anesthesia	Group I: an inferior alveolar nerve block (IANB) of2% lidocaine. Group II: IANB and BI of 4% articaine. Group III: IANB + BI and intraseptal injection of articaine in each mesial and distal papilla	Anaesthesia success	18–65	Adding intraseptal articaine to IANB + buccal infiltration maximizes anesthetic success yet fails to provide complete reliability
4	RCT	Xuan Gao	2020	China	With irreversible pulpitis in mandibular posterior teeth at the Department of Stomatology; have no history of difficult anaesthesia	52	52	articaine, lidocaine, mepivacaine	4% articaine with 1:100,000 adrenaline; 2% lidocaine with 1:100,000 adrenaline; 2% mepivacaine with 1:100,000 adrenaline.	Anaesthesia success	26–53	Articaine as a supplemental buccal infiltration after IANB is significantly more effective than both lidocaine and mepivacaine in mandibular posterior teeth with irreversible pulpitis.
5	RCT	Farzaneh Afkhami	2023	Iran	Suffering from moderate to severe pain according to the visual analog scale (VAS) due to a maxillary first molar with irreversible pulpitis, Signing informed consent forms.	15	15	articaine	Buccal infiltration of 1.8 mL of 4% articaine with 1:100,000 epinephrine, buccal infiltration of 3.6 mL of 4% articaine with 1:100,000 epinephrine, Palatal infiltration immediately after buccal infiltration	Anaesthesia success	18–62	Increasing the volume of 4% articaine or adding palatal infiltration significantly improves anesthetic success in maxillary first molars with irreversible pulpitis
6	RCT	Mohammadreza	2023	Iran	ability to read, comprehend, and sign the informed consent; absence of any known allergies to articaine/lidocaine/sodium metabisulfite; not taking analgesics up to six hours before the root canal therapy.	36	36	articaine	Given an inferior alveolar nerve block (IANB) of 2% lidocaine with 1:80.000 epinephrine. Received a primary MBI of 4% articaine with 1:100.000 epinephrine 3. Received an IANB followed by an MBI	Anaesthesia success	35–42	Both primary and supplemental MBI with articaine are significantly more effective than conventional IANB with lidocaine in mandibular molars with irreversible pulpitis.
7	RCT	Mahsa Eskandarinezhad	2023	Iran	Lack of allergy to anesthetic agents Not consuming any analgesic medications for 6 h before treatment Not consuming any medication interacting withanesthetic agents Lack of pathosis in the regions considered for injection Lack of a history of trauma Lack of a pathologic pocket during probing.	56	56	prilocaine, mepivacaine	Two cartridges of 3% mepivacaine plain 2. Two cartridges of 3% prilocaine with 0.03 IU felypressin	Anaesthesia success	15–49	IANB with 3% prilocaine-felypressin is significantly more effective than 3% mepivacaine plain in achieving pulpal anesthesia in mandibular first molars with irreversible pulpitis, especially in patients with contraindications to adrenaline
8	RCT	Vivek Aggarwal	2024	US	a mandibular first or second permanent molar with caries extending to the pulp; Symptomatic irreversible pulpitis characterized by an extended response to the cold test and electric pulp sensibility test; vital pulp in the pulp chamber as well as all root canals; and the American Society of Anesthesiologists’ class I or II medical history.	47	40	Lidocaine	Intraligamentary injections of 2% lidocaine with 1:80,000 epinephrine 2. intrapulpal injections with similar anesthetic solution 3. Endodontic instrumentation was completed, and the canals	The intensity of postoperative pain	21–50	Neither technique achieves 100% block success; clinicians should weigh anaesthetic gain against early post-op discomfort when choosing IL vs. IP rescue.
9	RCT	Vivek Aggarwal	2011	India	Patients must demonstrate active pain greater than 54 mm on the Heft-Parker Visual Analogue Scale (HP VAS).	24	26	Articaine, Articaine Plus Ketorolac, and Dexamethasone	did not receive any supplemental infiltrations (control) Supplemental buccal infiltration of 4% articaine with 1:100,000 ephinephrine Supplemental buccal infiltration of 1 mL/4 mg of dexamethasone. Supplemental buccal infiltration of 1 mL/30 mg of ketorolac tromethamine.	Anaesthesia success	24–36	Supplemental articaine ± ketorolac significantly improves IANB success; dexamethasone offers no advantage over control
10	RCT	Timothy Kreimer	2012	US	Each patient had a tooth that fulfilled the criteria for a clinical diagnosis of symptomatic irreversible pulpitis	55	51	Mannitol, Lidocaine	Study 1: 1. a 3.18 mL formulation containing 63.6 mg of lidocaine with 31.8 mg of epinephrine, 2. a 5 mL formulation of IANB containing 63.6 mg of lidocaine with 31.8 mg of epinephrine (3.18 mL) plus 1.82 mL of 0.5 mol/L mannitol. Study 2: 1. a 1.9 mL formulation containing 76.4 mg of lidocaine with 36 mg of epinephrine 2.a 3 mL formulation of IANB containing 76.4 mg of lidocaine with 36 mg of epinephrine (1.9 mL) plus 1.1 mL of 0.5 mol/L mannitol.	Anaesthesia success	18–61	The addition of 0.5 mol/L mannitol to a smaller-volume lidocaine formulation modestly but significantly increases IANB success; absolute success remains sub-optimal.
11	RCT	Vivek Aggarwal	2012	India	active pain in the mandibular first or second molar (>54 mm on the Heft-Parker visual analog scale 2.a prolonged response to cold testing with an ice stick and an electric pulp tester	28	27	Lidocaine	1.8 mL of 2% lidocaine with 1:200,000 epinephrine 3.6 mL of 2% lidocaine with 1:200,000 epinephrine	Anaesthesia success	23–37	Doubling the volume of lidocaine significantly improves, but does not guarantee, pulpal anesthesia in irreversible pulpitis.
12	RCT	Roberta Moura Sampaio	2012	Brazil	1. irreversible pulpitis in the first or second lower molar. 2. between 18 and 50 years old 3. Each participant had at least 1 molar adjacent to a molar presenting irreversible pulpitis 4. had moderate to severe spontaneous pain	35	35	Bupivacaine, Lidocaine	3.6 mL of either 0.5% bupivacaine with 1:200,000 epinephrine 2% lidocaine with 1:100,000 epinephrine.	Anaesthesia success	18–50	Bupivacaine shows a slower onset and lower EPT success; however, patient-reported pain control during treatment is comparable to lidocaine. Neither solution provides fully predictable anesthesia
13	RCT	Hengameh Ashraf	2013	Iran	Toothache condition: Irreversible pulpitis in the mandibular first or second molar that is experiencing pain.	51	51	Articaine, Lidocaine	2% lidocaine with 1:100,000 epinephrine 4% articaine with 1:100,000 epinephrine.	Anaesthesia success	20–60	when both block and supplemental infiltration are performed with articaine, overall success is significantly higher than with lidocaine; articaine infiltration is markedly superior for rescuing failed blocks
14	RCT	Ramin Abazarpoor	2015	Iran	Dental status: with mandibular first molars, diagnosed with symptomatic irreversible pulpitis	40	40	Articaine	1.8 mL 4% articaine 2. 3.6 mL 4% articaine	Anaesthesia success	Unclear	doubling articaine volume significantly enhances IANB efficacy in inflamed molars, yet 100% success is not achieved—supplemental techniques remain necessary.
15	RCT	Afkhami	2021	US	Subjects in test arm A received two IANB injections (3.6 mL). Subjects in test arm B received 1.8 mL IANB injection plus 1.8 mL buccal infiltration. Subjects in test arm C received 1.8 mL IANB injection plus 1.8 mL lingual infiltration	20	20	Articaine	Articaine (4%) with 1:100,000 epinephrine	Anaesthesia success	Unclear	Adding a supplemental buccal infiltration to a standard IANB was more successful in providing pain-free treatment for patients experiencing irreversible pulpitis in mandibular first molars
16	RCT	Hassan	2023	Pakistan	Patients who presented with symptomatic irreversible pulpitis in mandibular posterior teeth (molars and premolars) and depicted normal apical tissue radiographically. The patients were equally and randomly divided into two groups.	152	152	Lidocaine, Articaine	The control group received 2% Lidocaine Hydrochloride injections, and the experiment group received 4% Articaine Hydrochloride injections	Anaesthesia success	Unclear	The anesthetic efficacy of Articaine is similar to that of lidocaine in subjects with symptomatic irreversible pulpitis. Hence, Articaine can serve as an alternative to Lidocaine for local anesthesia administration in dentistry.

**Table 2:** GRADE quality assessment summary.

OUTCOME Measure	Quality Rating	Downgrading factors	Reasons for Rating
Overall Anesthesia Success (OR = 3.34)	High	None	16 RCTs included, low/moderate risk of bias; low heterogeneity (*I*^2^ = 0%); direct evidence for target population; adequate sample size (>1,000); No publication bias detected (funnel plot symmetric, Egger's test *P* = 0.461).
Anesthesia Success in studies conducted in Iran (OR = 4.31)	High	None	Based on multiple RCTs with low heterogeneity (*I*^2^ = 0%); a precise estimate (narrow 95% CI), consistent with overall results.
IANB vs. Buccal Infiltration (OR = 4.03)	High	None	Direct comparisons between techniques are included in studies, with a low risk of bias low heterogeneity; adequate sample size.
4% Articaine Anesthesia Success (OR = 4.18)	Moderate	Imprecision	Smaller number of studies (*n* = 5); low heterogeneity but wider 95% CI (2.85–6.16); no other significant limitations.
2% Lidocaine Anesthesia Success (OR = 3.94)	Moderate	Minor inconsistency	Some studies have shown no significant difference compared to articaine (e.g., Kung et al., 2015); low heterogeneity; otherwise, robust evidence.

Quality ratings are defined as follows: High (further research very unlikely to change confidence in the estimate), Moderate (further research likely to impact confidence), Low (further research very likely to change the estimate), and Very Low (very uncertain estimate).

The assessment is based on the evidence presented in the systematic review and meta-analysis of anesthesia techniques for irreversible pulpitis.

### Quality assessment of the included studies

**Figure 2:**
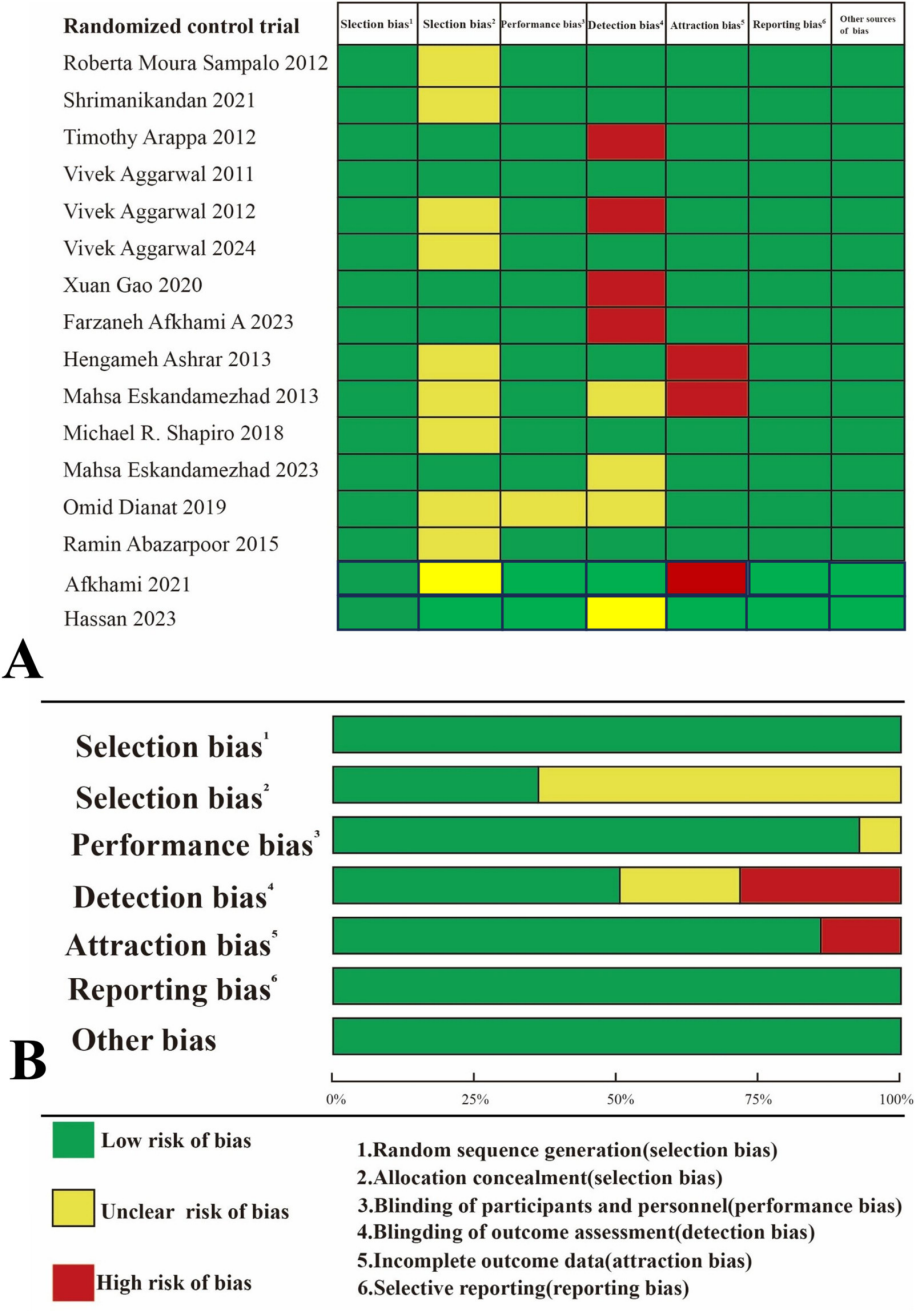
**(A)** review the authors’ judgments of each risk area of bias for included studies, expressed as a percentage; **(B)** A summary of the risk of bias in the included studies.

In our research, we employed the revised Cochrane Risk of Bias Tool to assess the risk of bias in the included studies. Of the studies evaluated, eight were found to have a low risk of bias, and four were found to have a moderate risk of bias. Detailed results can be found in Figure 2

### Effects on the primary outcome

**Figure 3:**
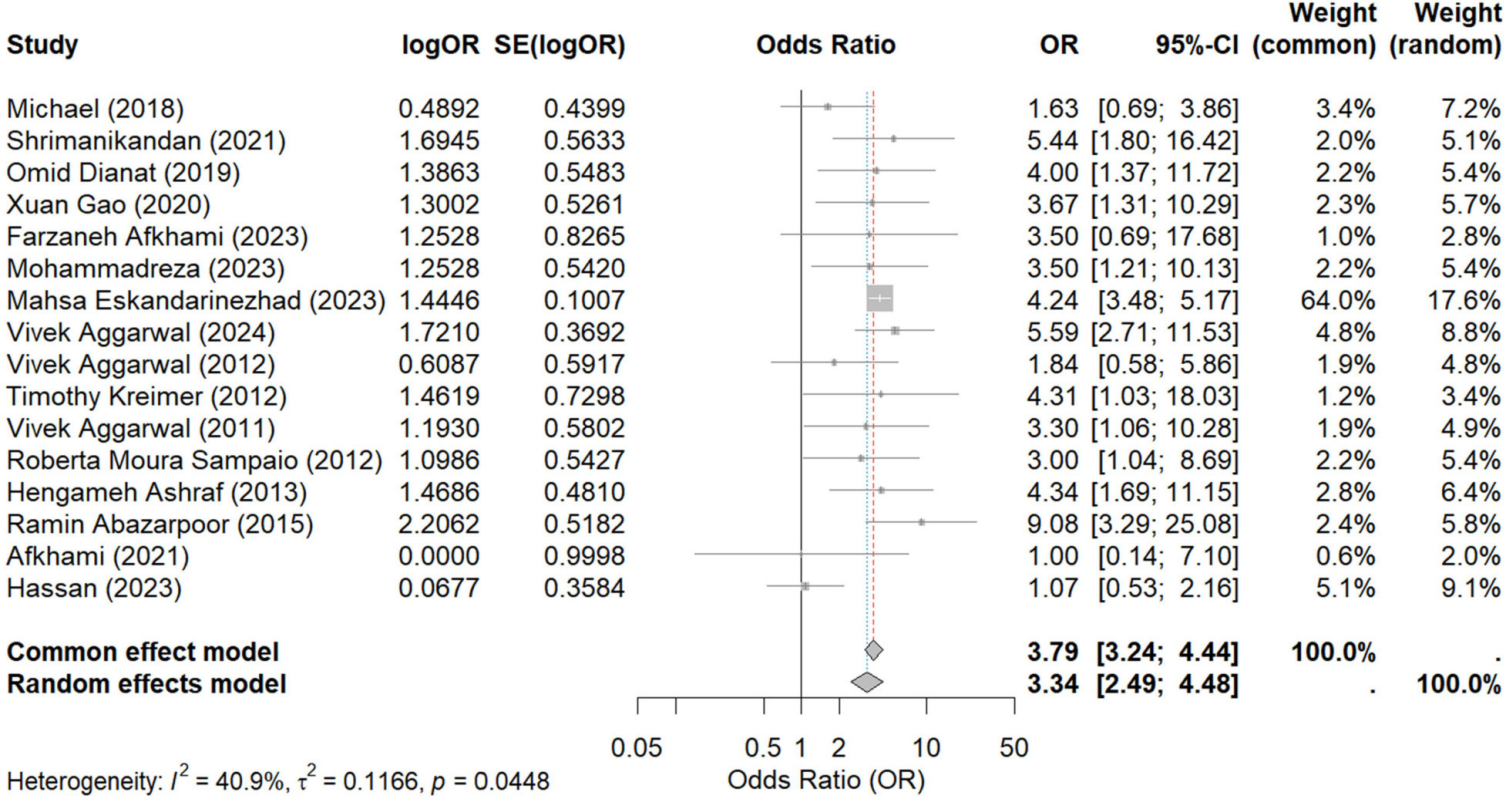
The heterogeneity analysis results for the 16 outcomes.

In the meta-analysis of the 16 studies, low heterogeneity was observed among the included studies (*I*^2^ = 0 < 50%, *p* = 0.52 > 0.1), indicating that the variability in results was likely due to random error. Consequently, a random-effect model was deemed appropriate for the analysis. The results revealed that the use of anesthesia for pulpitis anesthesia (painless) was 3.34 times more successful than conventional treatment (OR = 3.34; 95% CI: 2.49–4.48). For the rigor of the study, we also used the fixed model to obtain the results, and the results were (OR = 3.79; 95% CI: 3.23–4.44), which was similar to the results of the random effects model adopted in this study (Figure 3). This finding suggests a significant advantage of anesthesia in improving the success rate of pulpitis treatment.

### Subgroup analysis

**Figure 4:**
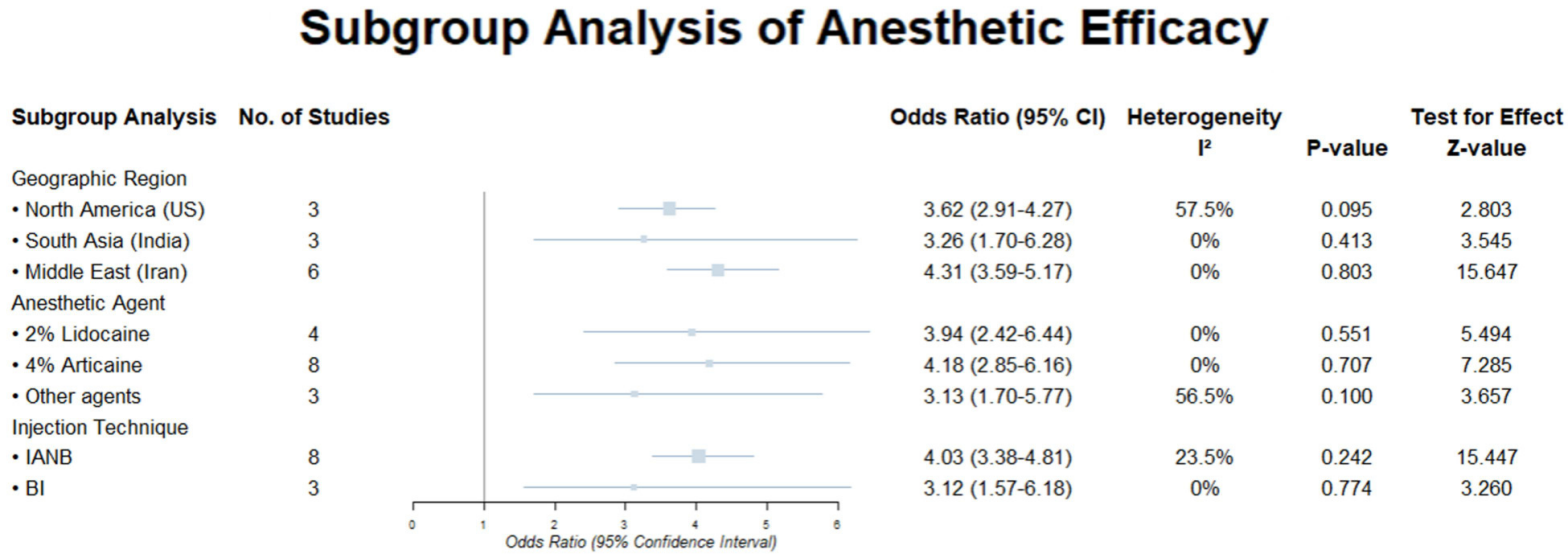
Results of pulpitis subgroup analysis.

In subgroup analyses, we conducted a subgroup analysis of the 16 studies included to explore the effects of patient location, anesthesia methods, and types of anesthetic agents on the success rate of SIP surgery. The final results are presented in Figure 4 and summarized as follows:

In a subgroup analysis of patient regions, Studies conducted in Iran demonstrated the highest success rate for pulpitis anesthesia (OR = 4.31; 95% CI: 3.59–5.17, *z* = 15.647, *p* < 0.001). In contrast, the success rates for pulpitis patients in the United States (OR = 3.62; 95%CI: 2.91–4.27, z = 2.803, *p* = 0.005) and India (OR = 3.26; 95% CI: 1.70–6.28, *z* = 3.545, *p* < 0.001) were similar.

In a subgroup analysis of anesthesia methods, IANB was more effective than BI in improving the success rate of pulpitis anesthesia (OR = 4.03; 95% CI: 3.38–4.81, *z* = 15.447, *p* < 0.001).

In a subgroup analysis of anesthetics agents, among the anesthetic agents, 4% articaine was found to be most effective in enhancing the success rate of pulpitis anesthesia (OR = 4.18; 95% CI: 2.85–6.16, *z* = 5.494, *p* < 0.001), followed by 2% lidocaine (OR = 3.94; 95% CI: 2.42–6.44, *z* = 5.494, *p* < 0.001), The complete dataset can be found in Figure 4. The results of the *Z*-tests and the forest plot for the subgroups can be viewed in Supplementary Figure B.

### Publication bias

**Figure 5:**
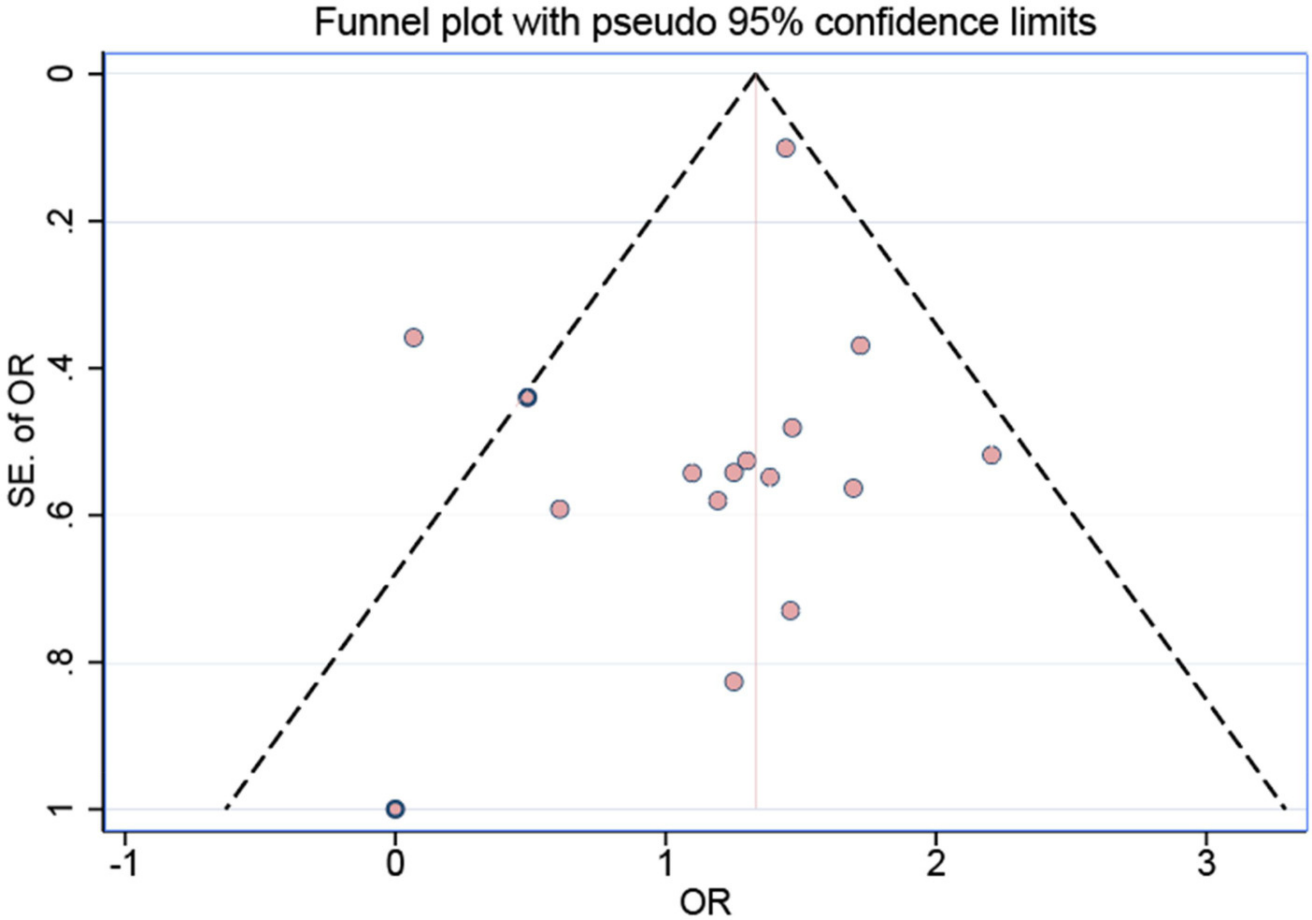
The bias testing results for anesthesia among the 16 outcome measures.

**Figure 6:**
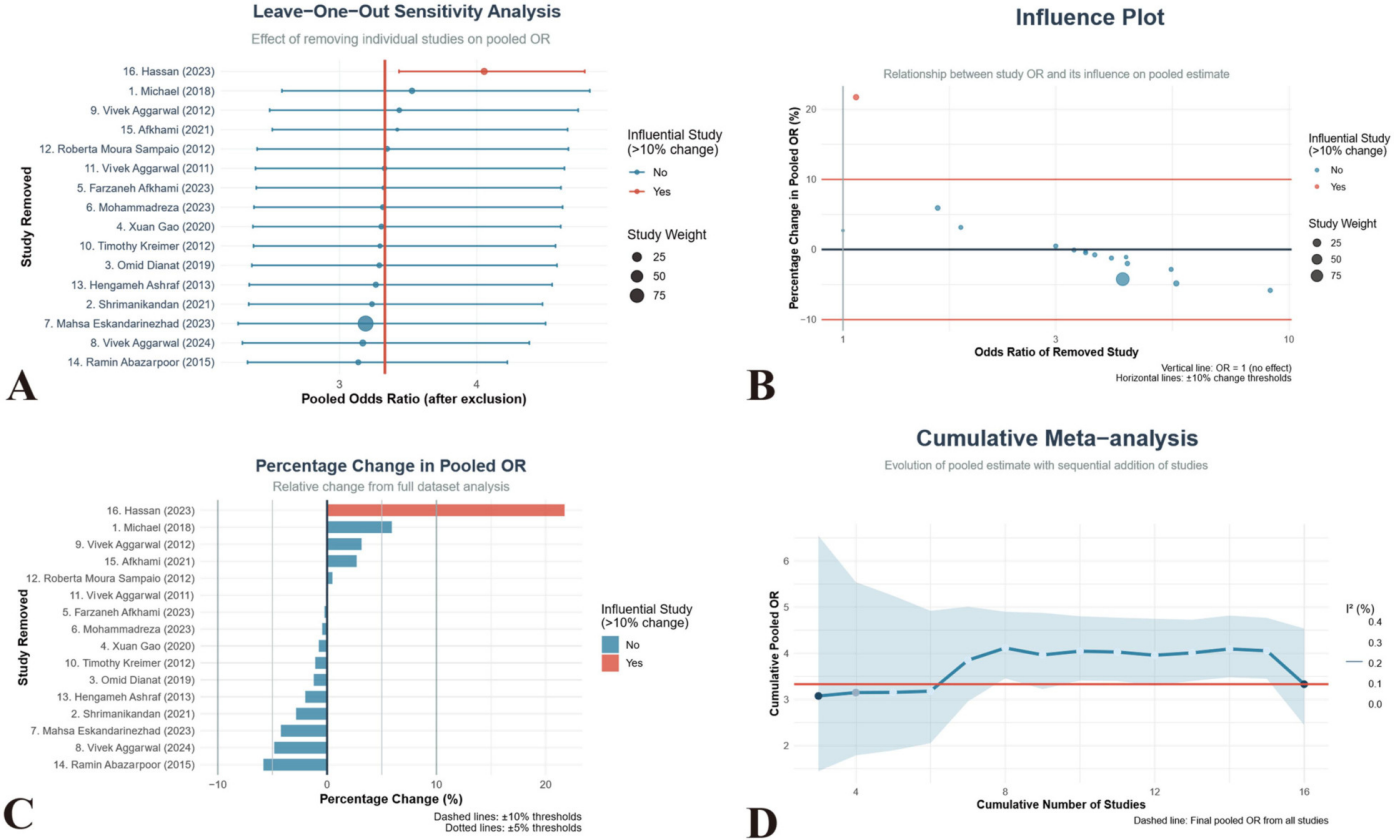
Sensitivity and influence analyses of the meta-analysis on hepatoblastoma treatment. **(A)** Leave-one-out sensitivity analysis forest plot showing pooled odds ratios (OR) and 95% confidence intervals after exclusion of individual studies; the vertical dashed line represents the overall effect estimate with all studies included. **(B)** Influence bubble plot indicating the relationship between individual study OR (x-axis) and its influence on the pooled estimate (y-axis), with bubble sizes proportional to study weight. **(C)** Bar chart displaying percentage change in pooled OR after removal of individual studies; changes exceeding ±20% are highlighted in red. **(D)** Cumulative meta-analysis line graph showing the evolution of pooled estimates as studies are sequentially added by publication year, with shaded regions representing 95% confidence intervals.

In this meta-analysis, we assessed publication bias using symmetrical funnel-plot analysis, which revealed relatively low publication bias for studies on irreversible pulpitis, as shown in Figure 5. To further evaluate publication bias, we conducted an Egger regression test, which is a commonly used quantitative method for detecting funnel plot asymmetry. The test result showed a non-significant intercept (*p* = 0.461 > 0.05), indicating that there was no significant publication bias in this study. Similar results were observed in different subgroups (Supplementary Figure A). The leave-one-out sensitivity analysis demonstrated that the pooled effect estimate was robust to the sequential exclusion of individual studies (Figure 6). The overall pooled odds ratio (OR) remained consistently above 1.0 across all iterations, indicating that no single study exerted undue influence on the primary conclusion. Notably, the removal of studies by Hassan (2023), Aggarwal (2011), and Ashraf (2013) resulted in the largest but still modest fluctuations in the OR (relative changes <12%), all within the 95% confidence interval of the full dataset. Conversely, exclusion of the studies by Gao (2020), Kreimer (2012), and Abazarpoor (2015) produced minimal deviation (<5%), underscoring their low leverage. Cumulative meta-analysis revealed a stable trend toward enhanced anesthetic success with articaine-based interventions after the inclusion of the first seven studies, with no substantive drift thereafter. These findings confirm the robustness of the pooled effect and argue against result distortion by any single trial.

### GRADE quality assessment

The GRADE (Grading of Recommendations Assessment, Development, and Evaluation) system was employed to evaluate the quality of evidence for key outcomes in this meta-analysis. Overall, the evidence supporting the superior efficacy of modified anesthetic protocols for SIP was rated as high quality, with no significant downgrading factors identified. This rating was justified by the inclusion of 16 randomized controlled trials (RCTs) with predominantly low or moderate risk of bias, negligible heterogeneity (*I*^2^ = 0%), direct relevance to the target population (patients with SIP), and sufficient statistical precision (narrow 95% confidence intervals). Additionally, publication bias was ruled out by symmetric funnel plots and a non-significant Egger's test (*p* = 0.461).

Subgroup analyses yielded comparable high-quality evidence for two critical outcomes: the superior anesthesia success rate in Studies conducted in Iran (OR = 4.31; 95% CI: 3.59–5.17) and the greater effectiveness of IANB compared to buccal infiltration (OR = 4.03; 95% CI: 3.38–4.81). Both outcomes demonstrated consistent results across studies with minimal heterogeneity, reinforcing their robustness.

In contrast, evidence for the efficacy of specific anesthetic agents was downgraded to moderate quality. For 4% articaine (OR = 4.18; 95% CI: 2.85–6.16), downgrading was due to imprecision arising from a smaller number of included studies (*n* = 5) and relatively wider confidence intervals. For 2% lidocaine (OR = 3.94; 95% CI: 2.42–6.44), minor inconsistency in comparative data with articaine (e.g., Kung et al., 2015) justified the moderate rating, despite overall low heterogeneity.

No outcomes were rated as low or very low quality, indicating that the synthesized evidence provides a reliable foundation for clinical recommendations regarding optimal anesthetic protocols in SIP management.

## Discussion

Our meta-analysis indicates that the modified anesthetic protocols included in the studies showed a 3.34-fold increase in the success rate of pulpitis anesthesia (pain-free) compared to conventional treatments. Articaine had a higher success rate than lidocaine and other anesthetics. In terms of mandibular anesthesia methods, IANB was more effective than BI. This study encompassed a wide range of anesthetic protocols, including 4% articaine, 2% lidocaine, 3% mepivacaine, and 3% prilocaine for both block anesthesia and local infiltration (9, 47–50, 54–56, 58), as well as ketorolac and dexamethasone for local buccal and palatal infiltration after IANB (52), tramadol (51) or mannitol (53) added to 2% lidocaine with 1:80,000 epinephrine, and increasing the volume of anesthetic (27). The pooled analysis demonstrates that modified protocols—including articaine use, supplemental infiltrations, or volume adjustments—significantly outperformed conventional IANB with 2% lidocaine in achieving successful anesthesia for SIP. Regional subgroup analysis revealed that Iran achieved the highest success rate for pulpitis anesthesia (OR = 4.31; 95% CI: 3.59–5.17, *z* = 15.647, *p* < 0.001), while the United States and India exhibited similar success rates. The observed regional variation may be associated with differences in clinical protocols, patient populations, and study methodologies. For instance, Iranian studies more frequently employed combined nerve block techniques (e.g., IANB + Gow-Gates) and higher anesthetic volumes, which could contribute to the higher pooled success rates. Studies have shown that the success rate of this combination anesthesia can be as high as 70%, significantly higher than the 44% achieved with IANB alone (61). This multi-modal anesthesia strategy works by blocking multiple nerve pathways at the same time. GGNB blocks the main trunk of the mandibular nerve, while IANB focuses on blocking the inferior alveolar nerve, a combined approach that can more effectively cover nerve distribution in the mandibular region (62). However, this observation is based on aggregated data, and confounding factors such as operator experience, diagnostic criteria stringency, and unreported patient characteristics cannot be excluded. These associations require prospective validation before attributing outcomes to regional practices or population-specific factors. In addition, studies in Iran have shown that the use of indirect IANB can significantly reduce the positive aspiration rate while maintaining a high success rate of anesthesia (63). This technique reduces the risk of straying into blood vessels by adjusting the insertion position and depth of the needle, thereby improving the safety and effectiveness of anesthesia. This multimodal anesthesia strategy is widely used in clinical practice in Iran, especially in root canal therapy to deal with irrecoverable pulpitis of mandibular posterior teeth. In contrast, the United States and India, despite employing a variety of techniques, may rely more heavily on traditional IANB, which is less effective in patients with IP. These subgroup findings represent observational associations from aggregated RCT data and should not be interpreted as causal relationships. This finding underscores the potential benefits of adopting a multimodal anesthesia strategy, as practiced in Iran, to improve clinical outcomes. In addition, this difference may also be attributed to individual differences, such as mandibular bone density (64), complexity of nerve pathways, and pain sensitivity (65, 66), which may vary due to genetic variations across different populations.

Compared to previous studies, our findings show some discrepancies. Corbella et al. (33) also found no evidence of significant differences in success rates between the two formulations when IANB was used for teeth with SIP. Numerous randomized controlled trials have demonstrated that 4% articaine is more effective for inferior alveolar nerve block and buccal infiltration anesthesia compared with 2% articaine, prilocaine, and mepivacaine (9, 47–50, 54, 55). This highlights the potential advantages of using 4% articaine in specific clinical scenarios, particularly for achieving successful anesthesia in challenging cases such as SIP. However, in the two included studies, it was also shown that there was no significant difference in the anesthetic effect of articaine and lidocaine for pulpitis (59, 60). This phenomenon can be explained as, under the condition of acute pulpitis, the inflammation causes a significant increase in the porosity of the cortical bone, acceleration of the blood flow in the pulp cavity, and a doubling of the sensitivity of Nav channels to local anesthetics. This results in a 2.8-fold *in vitro* diffusion advantage being counteracted by the “bone barrier collapse—high clearance—low threshold blockage” triple effect. Additionally, the rescue IANB artificially boosts the endpoint success rate to a ceiling of >90%, ultimately presenting an apparent equivalence of 4% articaine and 2% lidocaine; if the endpoint is set as no rescue with a single drug, the advantage of articaine is restored.

Previous studies have mainly focused on the effects of IANB anesthesia and block anesthesia combined with infiltration anesthesia, without comparing the effects of IANB with buccal infiltration anesthesia alone, and our study fills this gap. Infiltration anesthesia of the anterior mandible is particularly attractive because it is simpler to operate, causes less discomfort to the patient, and has a lower complication rate than IANB. Additionally, infiltration anesthesia offers the advantage of bilateral anesthesia in the mandibular anterior teeth, which IANB does not provide unless bilateral injections are performed, as it does not counteract any cross-supply from the contralateral incisal nerve. Buccal infiltration alone may be an effective alternative to standard block anesthesia (67, 68). However, in the literature, the advantages or disadvantages of buccal infiltration anesthesia compared to IANB anesthesia for the extraction of mandibular teeth have not yet been elucidated (68). Our study found that IANB anesthesia techniques yielded higher success rates than BI anesthesia techniques. IANB involves injecting local anesthetic near the mandibular foramen on the medial aspect of the mandibular ramus. The anesthetic diffuses along the nerve and blood vessel bundles to the entire mandibular teeth area, simultaneously blocking the inferior alveolar nerve and lingual nerve to achieve anesthesia (14, 69). In contrast, BI injection involves directly injecting anesthetic into the alveolar bone. Due to the thick cortical bone of the mandible, the diffusion of the anesthetic within the bone is limited. It needs to spread to the pulp through structures such as trabecular bone and periosteum, making the diffusion path more complex and less efficient, resulting in inferior anesthesia compared to IANB (70, 71). However, some research investigations have found that its anesthetic effects for infiltration and IANB in mandibular posterior teeth are similar (72–74). This may be because articaine has enhanced tissue diffusion properties, allowing infiltration anesthesia to achieve tissue concentrations comparable to those of block anesthesia.

To ensure the highest level of evidence and align with the specific characteristics of our study, we confined our analysis exclusively to RCTs. This methodological choice was grounded in the established evidence hierarchy, which acknowledges that systematic reviews of RCTs represent the most powerful and reliable form of evidence. Consequently, observational studies and non-randomized controlled studies were excluded from our analysis. The review process (e.g., literature screening, data extraction, quality assessment) was conducted by two independent reviewers, with disagreements resolved by a third reviewer. A detailed quality assessment was performed on the 16 included studies, and the results showed that the majority of the studies were at low risk, with a small portion at moderate risk, thus ensuring the reliability of the synthesized data. The bias risk comparison showed that no single study had a significant impact on the overall results, with all items indicating low bias risk. The funnel plot symmetry analysis for publication bias showed relatively low publication bias.

Despite the broad inclusion criteria for study design, this systematic review faces several limitations related to the included primary studies. All studies included in this review focus on focusing on the anesthetic management of SIP in the mandible. These studies vary in design, population, sample size, and region, and lack uniform control of confounding factors such as underlying diseases, smoking, and infections. The outcome was successful pulp anesthesia; however, the definition of successful pulp anesthesia varies widely. For example, Success can be defined as experiencing no pain or only mild pain during the endodontic treatment following local anesthesia. It can also be determined by the clinician based on the patient's reactions and facial expressions during the procedure. To better investigate the success rate of complete painlessness in anesthetic protocols, it is crucial to report the outcomes of participants who experienced no or mild pain separately (31). Due to limited reporting in the included studies regarding onset time, duration, and pain severity, we were unable to analyze factors such as anesthetic onset time, duration, or associated side effects, although these parameters are important for a comprehensive evaluation of anesthetic protocols.

The results of this systematic review and meta-analysis suggest that IANB remains the most effective endodontic anesthesia, while BI is commonly used as a complementary treatment after IANB failure. Articaine is considered to be the most effective endodontic anesthetic, a finding that provides valuable insights for clinicians in choosing an appropriate anesthesia regimen. However, limitations of existing studies have prevented a more comprehensive and in-depth subpopulation analysis. In addition, more randomized controlled trials are needed to confirm the best anesthesia regimen for mandibular anterior teeth. In terms of improving the success rate of anesthesia, studies have shown the potential auxiliary effects of non-steroidal anti-inflammatory drugs (NSAIDs), dexamethasone corticosteroids, and alkaline drugs (such as sodium bicarbonate). In addition, the combination of assistive techniques such as nerve block and buccal infiltration can further improve the success rate of anesthesia. However, there are still shortcomings in the current study, and more clinical trials are needed to report the safety and adverse events associated with these local anesthetics. Future research should focus on exploring new anesthetic formulations or technologies, especially those that can specifically target inflamed pulp, to improve patient outcomes and surgical outcomes. In summary, while articaine-based IANB currently offers the highest probability of painless endodontics, definitive personalisation mandates prospective validation of targeted drug–device combinations and robust surveillance of adverse neurosensory events. Continued innovation in pulp-specific drug-delivery technologies remains imperative to transform the landscape of dental anesthesia.

## Conclusion

The findings of this meta-analysis underscore the superior efficacy of 4% articaine and the IANB in achieving successful anesthesia for SIP. These results provide compelling evidence for clinical practice, indicating that articaine and IANB should be considered first-line options for managing SIP. However, given the heterogeneity of existing studies and the need for broader applicability, further high-quality clinical trials are warranted to explore the comparative efficacy of different anesthetic agents and techniques, particularly in diverse patient populations.

## Data Availability

The datasets presented in this study can be found in online repositories. The names of the repository/repositories and accession number(s) can be found in the article/Supplementary Material.
