# Supplementary material for: Evaluation of the effectiveness of local anesthesia approaches for symptomatic irreversible pulpitis: a systematic review and meta-analysis
Source: Front Dent Med. 2026 Jan 14;6:1679706. doi: 10.3389/fdmed.2025.1679706 (PMC12847389; doi:10.3389/fdmed.2025.1679706)

**Supplementary**

**Supplementary Table S1 PICOS framework**

| PICOS | P (Population) | Patients with symptomatic irreversible pulpitis receive endodontic treatment |
| --- | --- | --- |
|  | I (Intervention) | The main methods of anesthesia include buccal infiltration, tongue infiltration, intramuscular injection, interseptal injection, or a combination of these techniques |
|  | C (Comparison) | IANB |
|  | O (Outcome) | The success rate of anesthesia, defined as the proportion of patients achieving adequate pain control and procedural comfort during dental interventions |
|  | S (Study design) | RCTs |

**Supplementary Table S2 PRISMA 2020 Checklist**

| **Section and Topic** | **Item #** | **Checklist item** | **The location where item is reported** |
| --- | --- | --- | --- |
| **TITLE** | | |  |
| Title | 1 | Identify the report as a systematic review. | p. 1 |
| **ABSTRACT** | | |  |
| Abstract | 2 | See the PRISMA 2020 for Abstracts checklist. | p. 1 |
| **INTRODUCTION** | | |  |
| Rationale | 3 | Describe the rationale for the review in the context of existing knowledge. | p. 3-4 |
| Objectives | 4 | Provide an explicit statement of the objective(s) or question(s) the review addresses. | p. 3-4 |
| **METHODS** | | |  |
| Eligibility criteria | 5 | Specify the inclusion and exclusion criteria for the review and how studies were grouped for the syntheses. | p. 5 |
| Information sources | 6 | Specify all databases, registers, websites, organizations, reference lists, and other sources searched or consulted to identify studies. Specify the date when each source was last searched or consulted. | p. 5 |
| Search strategy | 7 | Present the full search strategies for all databases, registers, and websites, including any filters and limits used. | p. 5 |
| Selection process | 8 | Specify the methods used to decide whether a study met the inclusion criteria of the review, including how many reviewers screened each record and each report retrieved, whether they worked independently, and if applicable, details of automation tools used in the process. | p. 6 |
| Data collection process | 9 | Specify the methods used to collect data from reports, including how many reviewers collected data from each report, whether they worked independently, any processes for obtaining or confirming data from study investigators, and if applicable, details of automation tools used in the process. | p. 6 |
| Data items | 10a | List and define all outcomes for which data were sought. Specify whether all results that were compatible with each outcome domain in each study were sought (e.g. for all measures, time points, analyses), and if not, the methods used to decide which results to collect. | p.6 |
|  | 10b | List and define all other variables for which data were sought (e.g. participant and intervention characteristics, funding sources). Describe any assumptions made about any missing or unclear information. | p. 6 |
| Study risk of bias assessment | 11 | Specify the methods used to assess the risk of bias in the included studies, including details of the tool(s) used, how many reviewers assessed each study whether they worked independently, and if applicable, details of automation tools used in the process. | p. 6 |
| Effect measures | 12 | Specify for each outcome the effect measure(s) (e.g. risk ratio, mean difference) used in the synthesis or presentation of results. | p.7 |
| Synthesis methods | 13a | Describe the processes used to decide which studies were eligible for each synthesis (e.g. tabulating the study intervention characteristics and comparing against the planned groups for each synthesis (item #5)). | p. 7-8 |
|  | 13b | Describe any methods required to prepare the data for presentation or synthesis, such as handling of missing summary statistics, or data conversions. | p.7-8 |
|  | 13c | Describe any methods used to tabulate or visually display the results of individual studies and syntheses. | p.7-8 |
|  | 13d | Describe any methods used to synthesize results and provide a rationale for the choice(s). If meta-analysis was performed, describe the model(s), method(s) to identify the presence and extent of statistical heterogeneity, and software package(s) used. | p.7-8 |
|  | 13e | Describe any methods used to explore possible causes of heterogeneity among study results (e.g. subgroup analysis, meta-regression). | p.7-8 |
|  | 13f | Describe any sensitivity analyses conducted to assess the robustness of the synthesized results. | p. 7 |
| Reporting bias assessment | 14 | Describe any methods used to assess the risk of bias due to missing results in a synthesis (arising from reporting biases). | p. 8 |
| Certainty assessment | 15 | Describe any methods used to assess certainty (or confidence) in the body of evidence for an outcome. | p. 7-8 |
| **RESULTS** | | |  |
| Study selection | 16a | Describe the results of the search and selection process, from the number of records identified in the search to the number of studies included in the review, ideally using a flow diagram. | p. 8  Figure 1 |
|  | 16b | Cite studies that might appear to meet the inclusion criteria, but which were excluded, and explain why they were excluded. | Supplementary Table A |
| Study characteristics | 17 | Cite each included study and present its characteristics. | p. 8 |
| Risk of bias in studies | 18 | Present assessments of risk of bias for each included study. | p. 8  Figure 2 |
| Results of individual studies | 19 | For all outcomes, present, for each study: (a) summary statistics for each group (where appropriate) and (b) an effect estimate and it's precision (e.g. confidence/credible interval), ideally using structured tables or plots. | p. 8-9  Figures 3 |
| Results of syntheses | 20a | For each synthesis, briefly summarise the characteristics and risk of bias among contributing studies. | Table 1 |
|  | 20b | Present results of all statistical syntheses conducted. If meta-analysis was done, present for each the summary estimate and its precision (e.g. confidence/credible interval) and measures of statistical heterogeneity. If comparing groups, describe the direction of the effect. | p. 8-9 |
|  | 20c | Present results of all investigations of possible causes of heterogeneity among study results. | p. 8-9 |
|  | 20d | Present results of all sensitivity analyses conducted to assess the robustness of the synthesized results. | p. 8-9 |
| Reporting biases | 21 | Present assessments of risk of bias due to missing results (arising from reporting biases) for each synthesis assessed. | p. 9 |
| Certainty of evidence | 22 | Present assessments of certainty (or confidence) in the body of evidence for each outcome assessed. | p. 9 |
| **DISCUSSION** | | |  |
| Discussion | 23a | Provide a general interpretation of the results in the context of other evidence. | p. 10-11 |
|  | 23b | Discuss any limitations of the evidence included in the review. | p. 12-13 |
|  | 23c | Discuss any limitations of the review processes used. | p. 12-13 |
|  | 23d | Discuss the implications of the results for practice, policy, and future research. | p. 12-13 |
| **OTHER INFORMATION** | | |  |
| Registration and protocol | 24a | Provide registration information for the review, including the register name and registration number, or state that the review was not registered. | p. 1 |
|  | 24b | Indicate where the review protocol can be accessed, or state that a protocol was not prepared. | p. 1 |
|  | 24c | Describe and explain any amendments to information provided at registration or in the protocol. | No amendments. |
| Support | 25 | Describe sources of financial or non-financial support for the review, and the role of the funders or sponsors in the review. | Title page p. 1 |
| Competing interests | 26 | Declare any competing interests of review authors. | Title page p. 1 |
| Availability of data, code, and other materials | 27 | Report which of the following are publicly available and where they can be found: template data collection forms; data extracted from included studies; data used for all analyses; analytic code; any other materials used in the review. | Title page p. 1 |

**Table 1~5: Details of the Literature Search Strategy**

1. PubMed（Aug 17, 2024）

| **Search** | **Query** | **Results** |
| --- | --- | --- |
| #1 | "Pulpitis"[Mesh] | 3256 |
| #2 | ((((Pulpitides[Title/Abstract]) OR (Inflammation, Endodontic[Title/Abstract])) OR (Endodontic Inflammation[Title/Abstract])) OR (Endodontic  Inflammations[Title/Abstract])) OR (Inflammations, Endodontic[Title/Abstract]) | 893 |
| #3 | ("Pulpitis"[Mesh]) OR (((((Pulpitides[Title/Abstract]) OR (Inflammation, Endodontic[Title/Abstract])) OR (Endodontic Inflammation[Title/Abstract])) OR (Endodontic Inflammations[Title/Abstract])) OR (Inflammations, Endodontic[Title/Abstract])) | 4049 |
| #4 | "Anesthetics"[Mesh] | 89608 |
| #5 | ((((((((Anesthetic[Title/Abstract]) OR (Anesthetic Agents[Title/Abstract])) OR (Agents, Anesthetic[Title/Abstract])) OR (Anesthetic Drugs[Title/Abstract])) OR (Drugs, Anesthetic[Title/Abstract])) OR (Anesthetic Effect[Title/Abstract])) OR (Effect, Anesthetic[Title/Abstract])) OR (Anesthetic Effects[Title/Abstract])) OR (Effects, Anesthetic[Title/Abstract]) | 67469 |
| #6 | (((((((((Anesthetic[Title/Abstract]) OR (Anesthetic Agents[Title/Abstract])) OR (Agents, Anesthetic[Title/Abstract])) OR (Anesthetic Drugs[Title/Abstract])) OR (Drugs, Anesthetic[Title/Abstract])) OR (Anesthetic Effect[Title/Abstract])) OR (Effect, Anesthetic[Title/Abstract])) OR (Anesthetic Effects[Title/Abstract])) OR (Effects, Anesthetic[Title/Abstract])) OR ("Anesthetics"[Mesh]) | 132329 |
| #7 | "Randomized Controlled Trial" [Publication Type] | 631290 |
| #8 | ((("Pulpitis"[Mesh]) OR (((((Pulpitides[Title/Abstract]) OR (Inflammation, Endodontic[Title/Abstract])) OR (Endodontic Inflammation[Title/Abstract])) OR (Endodontic Inflammations[Title/Abstract])) OR (Inflammations, Endodontic[Title/Abstract]))) AND ((((((((((Anesthetic[Title/Abstract]) OR (Anesthetic Agents[Title/Abstract])) OR (Agents, Anesthetic[Title/Abstract])) OR (Anesthetic Drugs[Title/Abstract])) OR (Drugs, Anesthetic[Title/Abstract])) OR (Anesthetic Effect[Title/Abstract])) OR (Effect, Anesthetic[Title/Abstract])) OR (Anesthetic Effects[Title/Abstract])) OR (Effects, Anesthetic[Title/Abstract])) OR ("Anesthetics"[Mesh]))) AND ("Randomized Controlled Trial" [Publication Type]) | 143 |

1. Cochrane Library（Aug 11, 2024）

| **Search** | **Query** | **Results** |
| --- | --- | --- |
| #1 | MeSH descriptor: [Pulpitis] explode all trees | 581 |
| #2 | (Pulpitides ):ab,ti,kw OR (Inflammation, Endodontic ):ab,ti,kw OR (Endodontic Inflammation ):ab,ti,kw OR (Endodontic Inflammations ):ab,ti,kw OR (Inflammations, Endodontic ):ab,ti,kw | 111 |
| #3 | #1 OR #2 | 665 |
| #4 | MeSH descriptor: [Anesthetics] explode all trees | 19535 |
| #5 | (Anesthetic ):ab,ti,kw OR (Anesthetic Agents ):ab,ti,kw OR (Agents, Anesthetic ):ab,ti,kw OR (Anesthetic Drugs ):ab,ti,kw OR (Drugs, Anesthetic ):ab,ti,kw OR (Anesthetic Effect ):ab,ti,kw OR (Effect, Anesthetic ):ab,ti,kw OR (Anesthetic Effects ):ab,ti,kw OR (Effects, Anesthetic ):ab,ti,kw | 31799 |
| #6 | #4 OR #5 | 43082 |
| #7 | MeSH descriptor: [Randomized Controlled Trial] explode all trees | 37 |
| #8 | #3 AND #6 AND #7 | 0 |

1. Embase（Aug 11, 2024）

| **Search** | **Query** | **Results** |
| --- | --- | --- |
| #1 | Pulpitis | 4,898 |
| #2 | 'pulpitides':ab,ti OR 'inflammation, endodontic':ab,ti OR 'endodontic inflammation':ab,ti OR 'endodontic inflammations':ab,ti OR 'inflammations, endodontic':ab,ti | 8 |
| #3 | #1 OR #2 | 4,903 |
| #4 | Anesthetics | 35,238 |
| #5 | 'anesthetic':ab,ti OR 'anesthetic agents':ab,ti OR 'agents, anesthetic':ab,ti OR 'anesthetic drugs':ab,ti OR 'drugs, anesthetic':ab,ti OR 'anesthetic effect':ab,ti OR 'effect, anesthetic':ab,ti OR 'anesthetic effects':ab,ti OR 'effects, anesthetic':ab,ti | 85,763 |
| #6 | #4 OR #5 | 107,623 |
| #7 | Randomized Controlled Trial | 1,203,213 |
| #8 | #3 AND #6 AND #7 | 104 |

1. Scopus（Aug 11, 2024）

| **Search** | **Query** | **Results** |
| --- | --- | --- |
| #1 | Pulpitis | 5,641 |
| #2 | Pulpitides OR inflammation, endodontic OR endodontic inflammation OR endodontic inflammations OR inflammations, endodontic | 14,863 |
| #3 | #1 OR #2 | 23,054 |
| #4 | Anesthetics | 481,420 |
| #5 | anesthetic OR anesthetic agents OR agents, anesthetic OR anesthetic drugs OR drugs, anesthetic OR 'anesthetic effect OR effect, anesthetic OR anesthetic effects OR effects, anesthetic | 136,615 |
| #6 | (anesthetic OR anesthetic agents OR agents, anesthetic OR anesthetic drugs OR drugs, anesthetic OR 'anesthetic effect OR effect, anesthetic OR anesthetic effects OR effects, anesthetic) OR (Anesthetics) | 481,420 |
| #7 | Randomized Controlled Trial | 4,186,818 |
| #8 | #3 AND #6 AND #7 | 1,167 |

1. Web of Science（Aug 11, 2024）

| **Search** | **Query** | **Results** |
| --- | --- | --- |
| #1 | Pulpitis | 1,476 |
| #2 | Pulpitides OR inflammation, endodontic OR endodontic inflammation OR endodontic inflammations OR inflammations, endodontic | 696 |
| #3 | #1 OR #2  (https://webofscience.clarivate.cn/wos/woscc/summary/d2bb70f2-df71-49da-b6d8-0b7fdbcfe31f-015723e442/relevance/1) | 2,065 |
| #4 | Anesthetics | 47,132 |
| #5 | anesthetic OR anesthetic agents OR agents, anesthetic OR anesthetic drugs OR drugs, anesthetic OR 'anesthetic effect OR effect, anesthetic OR anesthetic effects OR effects, anesthetic | 47,271 |
| #6 | (anesthetic OR anesthetic agents OR agents, anesthetic OR anesthetic drugs OR drugs, anesthetic OR 'anesthetic effect OR effect, anesthetic OR anesthetic effects OR effects, anesthetic) OR (Anesthetics)  (https://webofscience.clarivate.cn/wos/woscc/summary/b7d54416-110f-41ad-a111-322fa0fd6590-01572439f2/relevance/1) | 47,305 |
| #7 | Randomized Controlled Trial | 416,069 |
| #8 | #3 AND #6 AND #7  (https://webofscience.clarivate.cn/wos/woscc/summary/c0645f24-d542-4a52-b744-7fea2df9096d-0157247450/relevance/1) | 63 |

**Figure A:** Bias testing results among multiple subgroups


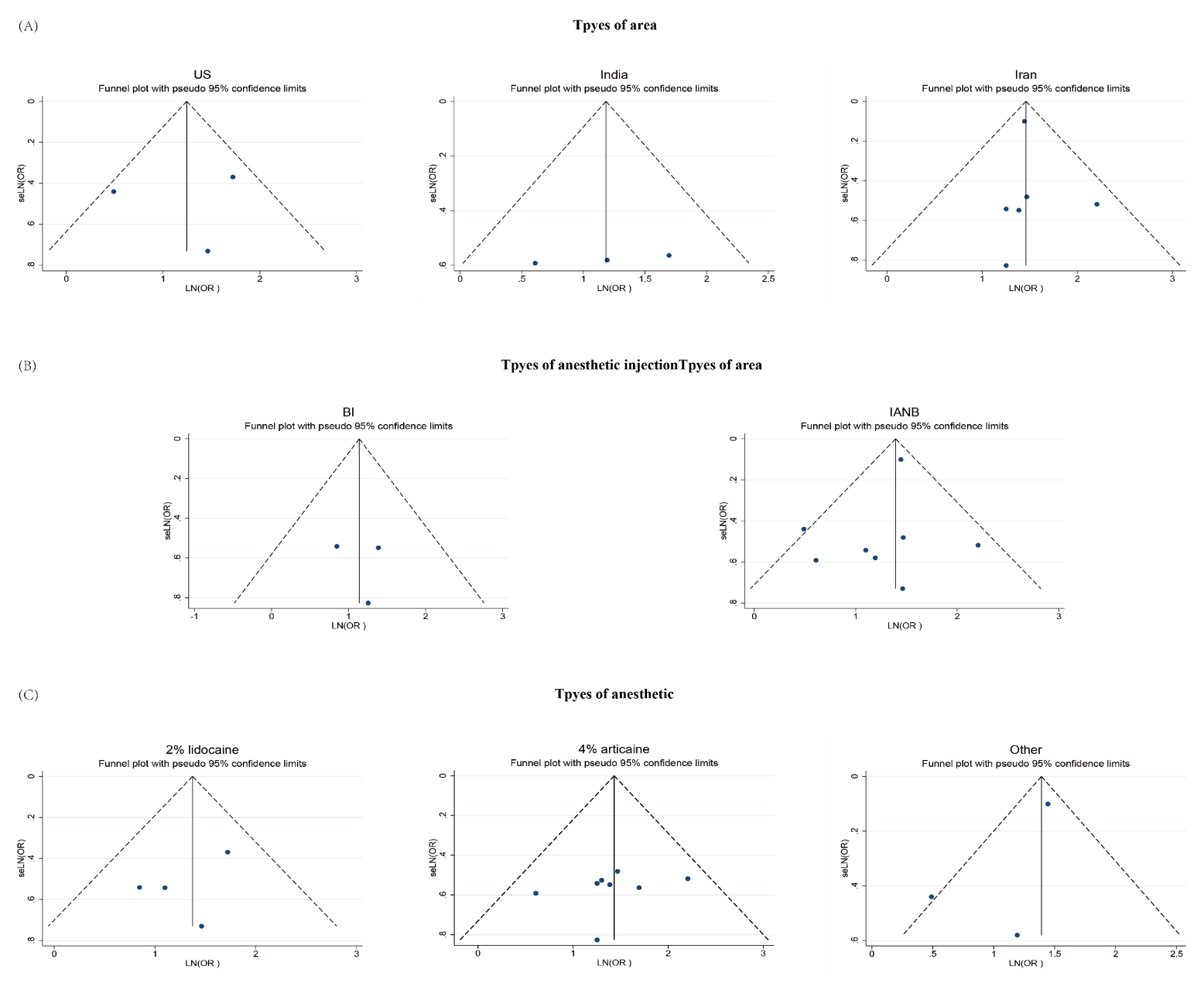


**Figure B:** The heterogeneity test results (forest plot) for each subgroup and the p-values for the z-test


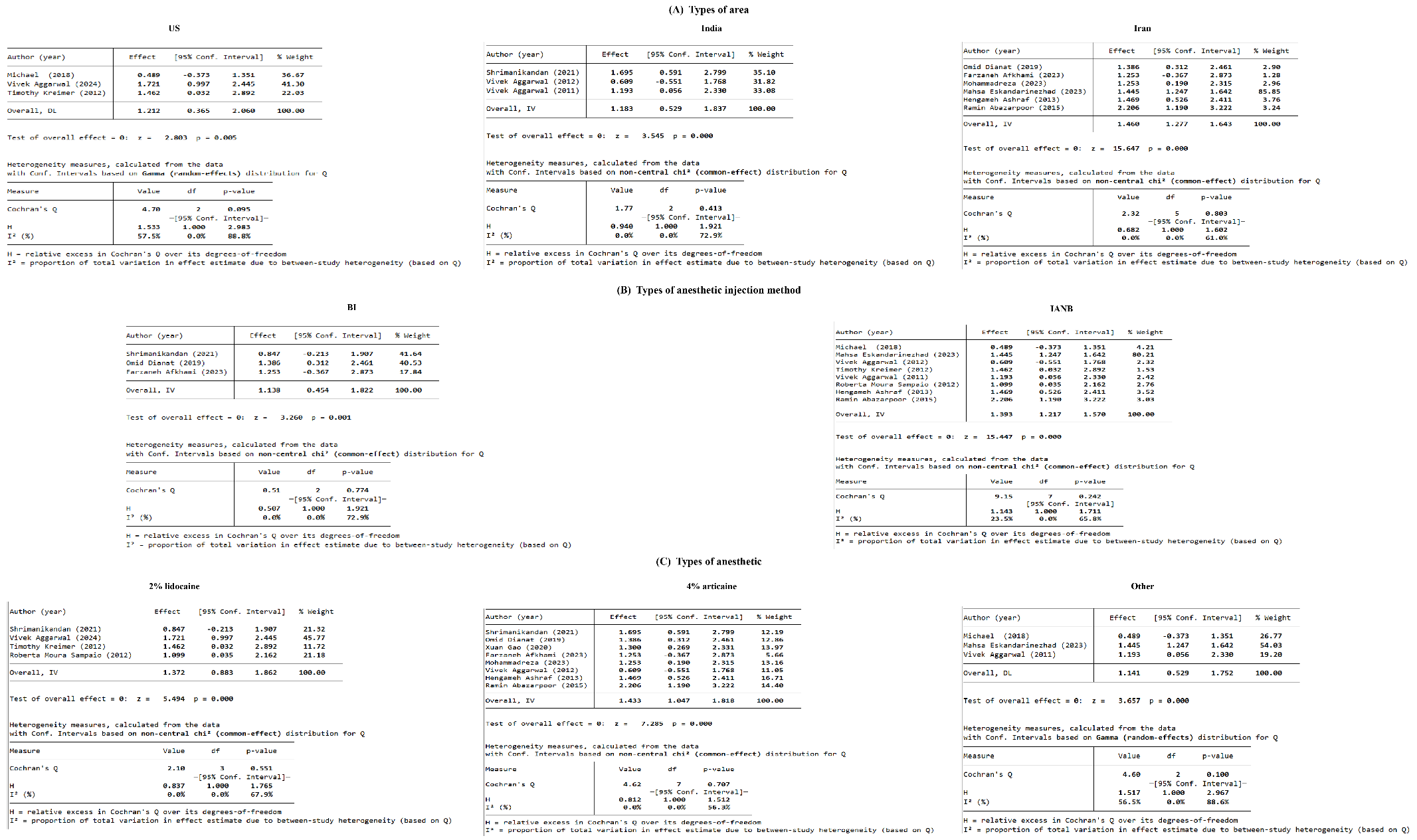

Supplement: Supplementary file 1 [file Table1.docx]
